# Awareness Regarding Causes of Infertility Among Out-patients at a Tertiary Care Hospital in Karachi, Pakistan

**DOI:** 10.7759/cureus.7685

**Published:** 2020-04-16

**Authors:** Hamza M Ahmed, Mohammad Khan, Farah Yasmin, Haris Jawaid, Hiba Khalid, Anum Shigri, Faryal Nawaz, Choudhary A Hasan

**Affiliations:** 1 Internal Medicine, Aga Khan University, Karachi, PAK; 2 Internal Medicine, Civil Hospital Karachi, Dow University of Health Sciences, Karachi, PAK; 3 Cardiology, Civil Hospital Karachi, Dow University of Health Sciences, Karachi, PAK; 4 Oncology, Dow University of Health Sciences, Karachi, PAK; 5 Internal Medicine, Dow University of Health Sciences, Karachi , PAK; 6 Surgery, Dow Medical College, Karachi, PAK; 7 Internal Medicine, Dow University of Health Sciences, Karachi, PAK

**Keywords:** infertility, awareness, knowledge, developing country

## Abstract

Introduction: Infertility is the inability of a couple to achieve pregnancy within 12 months of sexual intercourse without the use of contraceptives. The Pakistani population, belonging to a low-middle income country, has a high prevalence of infertility due to a low knowledge and awareness regarding its causes, and lack of healthcare-seeking behavior for this medical issue. The prevalence of infertility in Pakistan is reported as 22%, with primary infertility accounting for 4% of the total cases. This leads to psychological trauma among women as societal norms equate infertility with failure on a personal, emotional, and social level. In this study, we aimed to assess among this population the general awareness regarding infertility and its causes; and identify any key knowledge gaps pertaining to the subject.

Methods: A cross-sectional study was carried out between June 2019 and November 2019, at a public hospital (Civil Hospital Karachi) in Karachi, Pakistan. Convenience sampling technique was used to collect data from adult participants (older than 18 years) via an interview-administered questionnaire. The questionnaire was based on the Cardiff Fertility Knowledge Scale (CFKS) and assessed the knowledge regarding causes of infertility such as smoking, healthy lifestyle, contraceptives, genital tract infections among others. We also determined the association between socio-demographic variables with mean knowledge scores. Data were analyzed using Statistical Package for Social Sciences (SPSS), version 20.0.

Results: The majority of the participants were married (n=342, 68.8%) and more than half were unemployed (n=259, 52.1%). Approximately, two-quarters (n=250, 50.3%) did not believe that equal proportions of males and females contribute to infertility. The mean knowledge score of our study population was 12.95 ± 2.48 points. An overwhelming proportion of the participants (n=326, 65.6%) falsely believed that the usage of the intra-uterine device contributes to infertility. Additionally, more than half of the responders (n=278, 55.9%) incorrectly believed that a male achieving erection is an indication of fertility. Education (p=0.019), vehicle ownership (p=0.018), and marital status (p=0.031) were the only demographic factors that showed significant differences with mean knowledge scores.

Conclusion: Awareness regarding the causes of infertility among the general population was found to be inadequate. Emphasis on targeted fertility education, in association with general public awareness programs regarding its causes and risk factors may help mitigate this problem by potentially reducing the prevalence of this condition, and increasing the number of affected individuals who seek medical care in a timely fashion.

## Introduction

Infertility is a public reproductive health problem defined as an inability to conceive after one year of regular unprotected sexual interaction. It can be classified into primary and secondary infertility. Primary infertility refers to the inability to conceive after 12 months of unprotected sexual intercourse with no previous conceptions. However, secondary infertility arises when couples bearing previously conceived children are now unable to conceive [1]. Infertility is a global phenomenon affecting approximately 48.5 million couples in 2010 worldwide [2]. According to the Centers for Disease Control and Prevention (CDC) reports, the prevalence of infertility in a married woman aged 15-49 years is estimated to be 8.8% in the United States between 2015 and 2017 [3]. The incidence of infertility is higher in developing countries due to a lack of basic knowledge about the causes of infertility and the potential treatment required. The prevalence of infertility in Pakistan was reported as 22%, with primary infertility accounting for 4% of the affected individuals [4].

Infertility or subfertility is a disconcerting finding for couples, and often has severe ramifications in their daily lives such as issues in marital adjustment and psychological distress (including anxiety and depression). In addition to the general marginalization and stigmatization of the female sex, there is a markedly stubborn attitude amongst men concerning getting tested for infertility and a general refusal to acknowledge their role in infertility problems faced by the couple [5]. The infertile woman exhibits significantly higher psychopathology in the form of tension, hostility, anxiety, depression, self-blame, and suicidal ideation [6]. For instance, a study conducted by Daar and Merali in Andhra Pradesh, India reported 70% of women who experienced infertility to be punished with physical violence for their childlessness [7]. Similarly, another study conducted by Hakim et al. in Karachi to evaluate the psycho-social consequences of secondary infertility revealed that more than two thirds (67.7%) of women, who were unable to give live births or give birth to sons had marital conflicts. Additionally, these women were reported to receive divorce threats (20%) and were forced to return to their parent’s homes (26%) by their in-laws or husbands [8].

Although the incidence of infertility is on the rise, knowledge regarding risk factors of infertility is lacking in many parts of the world. A study conducted to evaluate infertility awareness among women visiting a tertiary fertility clinic revealed approximately three-quarters (76%) of the women to have an inadequate understanding of the fertile period in their menstrual cycle and timely infertility management [9]. It has been observed that although couples have a basic knowledge of factors affecting fertility, they remain unaware of the impact that advancing age has on a women’s fertility [10]. Regarding alternative strategies for conception in couples with infertility, a study showed that while people are aware of options such as in-vitro fertilization (IVF), around 39% grossly overestimated its efficacy [11].

Studies exploring the behaviors, perceptions, and practices regarding infertility or certain treatment options have been carried out in countries like Iran [1]. However, to the best of our knowledge, no such study has been conducted in a Pakistani population. Hence, the primary objective of our study was to assess the general knowledge and awareness in this population regarding infertility and its causes. Our secondary aim was to compare the knowledge scores between the two sexes, and identify specifically those domains of knowledge responsible for any dichotomy in scores observed between males and females.

## Materials and methods

A cross-sectional study was conducted between June 2019 and November 2019, at a tertiary care hospital in Karachi, Pakistan. A sample size of 471 was calculated considering the population size of 1,000,000 with an anticipating frequency of 50% and a 97% confidence interval. The sample size was increased to 497 to have a better representation of the general population. The study population included both men and women aged 18 years or older visiting the various specialized care, or primary care outpatient clinics. Individuals who were healthcare professionals, or were diagnosed with infertility or subfertility were excluded from this study as they were more likely to be better informed on this subject compared to the general population. A convenience sampling technique was implemented using an interview-administered questionnaire. Interviewers were fluent in English, as well as Urdu (the national language), and were trained to administer the questionnaire to eliminate interviewer bias. They were asked to wear identical lab coats, offer prepared explanations for questions, and offer the same amount of time to each person. 

The principal investigator explained the purpose of the study and the methodology to all participants included in the study. Participants were explained their right to freely choose whether or not to participate in the study, and that they could withdraw from it at will and at any time. Those who agreed to participate were also assured that their confidentiality and anonymity would be maintained to obtain as accurate answers as possible. To avoid the possibility of a nonresponse bias, the questionnaire was clear, kept as concise as possible, and did not take much time to complete. Finally, the participants were also asked to sign a written consent form along with verbal consent before including them in the study. An inclusive pilot study was also conducted on 6% of the calculated sample size to test the validity of our questionnaire, and any necessary modification to the questionnaire was made during its course.

A three-section standardized questionnaire was designed to collect data for this study. The first section inquired about the socio-demographic characteristics of the participants including age, gender, level of education, vehicle ownership, and marital status. The second section asked the participants regarding their general perception and if they believed that male and female factors contribute to infertility. The third section consisted of 22 questions to assess the knowledge pertaining to fertility facts, risks, and myths. These questions were extracted from the Cardiff Fertility Knowledge Scale (CFKS; Bunting et al., 2013) while others were formulated based on a review of the literature related to infertility and sexual health knowledge (16 and 21). These questions referred to facts (e.g. a woman is less fertile after the age of 36 years), risks (e.g. smoking decreases female fertility), and myths (e.g. if a man produces sperm, he is fertile). All items were rated on a three-point scale of ‘true’, ‘false’, or ‘do not know’. The items were combined into a composite correct variable, where one point was awarded for each correctly identified fact, risk or myth, with the maximum overall score being 22 points. The ‘do not know’ response was coded as incorrect. Additionally, the questionnaire was reviewed by two infertility specialists for validity and reliability.

Grading of the questions was carried out by two separate investigators (MOK and HM) and any discrepancies were resolved by mutual discussion or the involvement of a third investigator (FY). Only completely filled questionnaires were included in the analysis, and no imputation methods were implemented to account for missing data. Data were analyzed using Statistical Package for Social Sciences (SPSS), version 20.0. Categorical variables were expressed as frequencies and percentages, while continuous variables were expressed as means and standard deviation. Analysis of variance (ANOVA) was employed to compare mean knowledge scores with each socio-demographic factor and to determine if an association existed between them and participants’ knowledge about infertility. A value of p<0.05 was considered significant in all statistical analyses conducted in the study.

## Results

Baseline characteristics of the participants 

A total of 497 people participated in this study out of which more than three-fifths, (n=328, 66%) were females. A huge proportion (n=342, 68.8%) were married while more than half (n=286, 57.5%) had children. Slightly more than one-third (n=166, 33.4%) aged between 41 and 60 years with very few (n=49, 9.8%) being older than 60 years of age. Additionally, most participants completed some form of secondary education. An equal proportion (n=100, 20.1%) received O-Levels/Matriculation and A-levels/Intermediate degrees. A vast majority of participants in this study were unemployed (n=259, 52.1%), while more than one-third (n=205, 36.2%) were employed and a small proportion (n=21, 4.2%) had their private businesses. Less than half (n=220, 44.3%) of the participants owned one motor vehicle at the time of this study whereas approximately two-fifths (n=194, 39%) did not own one. Ownership of the vehicle was used as a surrogate marker of socio-economic status.

General perception about infertility 

Nearly two-quarters (n=250, 50.3%) did not believe that equal proportions of males and females contribute to infertility. Almost one-third (n=137, 27.6%) falsely believed that mostly female factors with some male factors contribute to infertility. Less than a quarter (n=87, 17.5%) reported only female factors to be responsible, followed by a small proportion (n=26, 5.2%) believing male factor to be the only factor responsible for infertility.

Knowledge of causes and factors contributing to infertility 

The mean knowledge score of our study population was 12.95 ± 2.48 points. Table 1 shows that the role of blocked fallopian tubes on infertility was correctly answered by more than three-quarters (n=436, 88%) of our cohort. However, the role played by contraceptives in causing infertility was correctly answered by a very small proportion (n=137, 28%) of our sample population. An overwhelming proportion of the participants (n=326, 65.6%) falsely believed that the usage of the intra-uterine device contributes to infertility. Additionally, more than half of the responders (n=278, 55.9%) incorrectly believed that a male achieving erection is an indication of fertility. Slightly more than two-quarters of the participants (n=285, 57.3%) did not acknowledge the influence of obesity on infertility. More than half of the participants (n=271, 54.5%) were unaware that smoking decreases male fertility. Also, more than one-third (n=181, 36.4%) did not acknowledge the fact that sexually transmitted diseases (STDs) can lead to infertility.

**Table 1:** The proportion of participants with correct responses for each knowledge question. PID, pelvic inflammatory disease

Knowledge assessment questions	n (%)
Fertility in women decreases after 36 years of age	338 (68)
A couple is classified as infertile if they did not achieve pregnancy after one year of regular sexual intercourse without using contraception	239 (48)
Smoking decreases female fertility	321 (65)
Smoking decreases male fertility	225 (45)
10% couples are infertile	312 (63)
If a man produces sperm, he is fertile	350 (70)
These days a woman in her 40s has a similar chance of getting pregnant as a woman in her 30s	266 (54)
Having a healthy lifestyle makes you fertile	361 (73)
Mumps after puberty can cause fertility problems in men	307 (62)
A woman who never menstruates is still fertile	380 (76)
If a woman is overweight by more than 13 kg, then she may not be able to get pregnant	212 (43)
Ability to achieve an erection in men indicates fertility	214 (43)
People who have had a sexually transmitted disease are likely to have reduced fertility	313 (63)
Having abnormal menses can cause infertility	316 (64)
In females, having blocked tubes can cause infertility	436 (88)
History of genital tract infections such as PID in females can cause infertility	374 (75)
Previous use of contraceptive pills by females can contribute to infertility	137 (28)
Previous use of the intrauterine device by female contributes to infertility	169 (34)
Jinns/supernatural phenomenon cause infertility	342 (69)
Regular exercise can cause infertility	355 (71)
Psychological stress can cause infertility	306 (62)
An equal proportion of male and female factors contribute to infertility	247 (50)

Correlation of socio-demographic factors with infertility knowledge 

There was no significant association between socio-demographic factors including gender (p=0.513), age (p=0.656), having children (p=0.166), and employment (p=0.978) with mean knowledge scores. Education (p=0.019), vehicle ownership (p=0.018), and marital status (p=0.031) were the only demographic factors that showed significant differences with mean knowledge scores. Referring to Table 2, it can be observed that participants with undergraduate and higher qualifications showed better knowledge regarding infertility with Master’s degree holders having the highest mean score of 13.69 ± 2.71 points. Participants who owned more than one vehicle had a higher mean knowledge score of 13.36 ± 2.27 points than others. Single participants had a significantly higher mean knowledge score of 13.34 ± 2.29 points than married or divorced participants.

**Table 2:** Co-relation of mean knowledge scores with socio-demographic characteristics. *p < 0.05

Characteristics	n (%)	Mean knowledge scores	p-value
Gender			0.513
Male	169(34)	12.95 ± 2.48	
Female	328(66)	12.84 ± 2.59	
Age (years)			0.656
18-25	132(27)	13.15 ± 2.45	
26-40	150(30)	12.86 ± 2.67	
41-60	166(33)	12.74 ± 2.54	
61-80	49(10)	12.67 ± 2.52	
Level of education			0.019*
No formal education	68(14)	12.27 ± 2.53	
Primary school	85(17)	12.63 ± 2.58	
Matric/O-levels	100(20)	13.16 ± 2.78	
Inter/A-levels	100(20)	12.5 ± 2.33	
Undergraduate	88(18)	13.43 ± 2.26	
Graduate (masters)	39(8)	13.69 ± 2.71	
Post-graduate	13(3)	12.53 ± 2.4	
Others	4 (1)	12 ± 3.55	
Employment status			0.978
Unemployed	259(52)	12.89 ± 2.53	
Employed	205(41)	12.85 ± 2.53	
Retired	12(2)	12.91 ± 3.17	
Private business	21(4)	13.09 ± 2.5	
Vehicle ownership			0.018*
None	194(39)	12.5 ± 2.43	
One	220(44)	12.99 ± 2.74	
More than one	83(17)	13.36 ± 2.27	
Marital status			0.031*
Married	342(69)	12.73 ± 2.6	
Divorced	19(4)	12.21 ± 3.02	
Single	136(27)	13.34 ± 2.29	
Children			0.166
Yes	286(58)	12.74 ± 2.58	
No	211(42)	13.06 ± 2.51	

## Discussion

The findings of our observational study demonstrated a significant lack of knowledge, as indicated by the low mean knowledge score among the sample population frequenting the out-patient clinics of a tertiary care hospital in our country. Considering only the correctly identified causes and risk factors of infertility, it is clear that the knowledge among the general population pertaining to the aforementioned is inadequate. This is not surprising as published literature showed limited knowledge among people about infertility worldwide. A global study carried out, involving over 17,000 people, highlighted that adequate knowledge regarding fertility and reproductive biology was insufficient [12]. Additionally, our results help highlight that more than half of our sample population was unaware that no single sex is exclusively responsible for infertility, with almost one-third believing female factors to be the only factor responsible for infertility. This is one of the reasons why motherhood is often the only way for women to enhance their status and prestige within the family and community and those who failed to conceive are treated with humiliation and shame. This false belief then eventually contributes to the life-threatening physical violence and psychological consequences experienced by women in developing countries further bolstering the fact that infertility significantly influences both the social and economic well-being of women [6-8].

From our study, it was also apparent that people were unaware of the biological aspects of conception including the effect of abnormal menses and the steep decline in fertility potential after the age of 34-35 years [13]. Though it was encouraging to observe that out of the 22 questions inquired to assess the knowledge of infertility, most of the participants (88%) were able to answer correctly that blocked fallopian tubes cause infertility while more than a third (36%) of the respondents did not recognize irregular menstruation as a cause of infertility. Furthermore, although sexually transmitted infections (STIs) are significant contributors to tubal damage and infertility, it was surprising to observe that only a small proportion (37%) could recognize STIs as a cause of childlessness. However, this proportion was greater than that observed in a study conducted in rural Ghana which revealed that less than 2% of participants referred to STIs as a cause of infertility [14]. The differences in the findings of the study could be attributed to the varying levels of education and development of the two countries. Also, as our study was conducted in an urban area, the participants had higher levels of education that was potentially a key factor responsible for the better levels of knowledge among the participants.

Moreover, the findings of this study show that knowledge and awareness regarding infertility and its risk factors are significantly influenced by the participants' level of education. This is because schools are identified as the most common source of sexual health information for adolescents, enabling them to obtain factually correct and useful knowledge regarding infertility that will aid them for the remainder of their lives [15]. Health initiatives such as Centers for Disease Control’s Infertility Prevention Project-STD and the American Society for Reproductive Medicine’s Protect Your Fertility initiatives target the United States adolescent population [16]. It is essential that such initiatives be taken by the health authorities in Pakistan to narrow the gap in infertility awareness between different levels of education.

Another interesting finding of our survey was the association of oral contraceptive pills and infertility as very few answered correctly regarding the role of oral contraceptives (OCP) in causing infertility. A similar result for OCP was seen in a study in which half of the participants believed that OCP causes infertility [17]. This belief contributes to the underutilization of these contraceptive methods for the fear that they may lead to infertility and adds to the issue of high parity in developing countries [18]. It is essential that contraceptive education is made a part of the basic formal education (at the secondary or high school level) to eliminate such myths.

Furthermore, research has clearly established the considerable negative impact of tobacco consumption on fertility in both males and females, pre and postconception [19]. Despite this fact, more than half our population did not believe that infertility is often attributable to smoking. Aryanpur et al. in his study showed brief counseling to be an effective intervention for smoking cessation by reporting successful cessation of smoking following the provision of an educational package by the treating physician in the infertility clinics [20]. Moreover, less than three-fifths of our cohort did not identify obesity as a potential cause of infertility. Obesity affects fertility in both men and women. In women, obesity is associated with increased risks of menstrual miscarriage, pregnancy complications, dysfunction and anovulation, [21] while spermatogenesis is impaired by obesity in men [22].

In our survey, one-third of our study population considered Jinns/supernatural powers to be responsible for infertility. These findings could be attributed to the cultural beliefs and values of the wide variety of socio-cultural and ethnic groups in Pakistan. These trends in our study are also similar to a previously conducted study by Bunting and Boivin [12]. We believe this is because people belonging to different countries worldwide accumulate many misconceptions regarding reproductive health due to fertility being considered as a taboo subject. The beliefs in evil forces and supernatural powers as a cause of infertility are still prevalent in many parts of the world. Women who are unable to conceive are considered to be possessed by evil spirits with infertility being a punishment from God. This leads them to seek treatment from faith healers thus negatively affecting its medical management, as evidenced by a study showing that 35.2%-44.2% of outpatients in Saudi Arabia considered a Sheikh (faith healer) as a treatment option for their infertility [17].

Additionally, our study showed no such significant relation of gender difference and knowledge of infertility indicating that both men and women lack knowledge about the effects of smoking, exercise, psychological stress, and other lifestyle factors on fertility potential. This finding is in agreement with the findings of Bunting and Boivin [12]. However, certain studies show that there are differences in knowledge between men and women about infertility, with women having more knowledge [18, 23]. A more thorough evaluation of the role of gender on infertility knowledge can be carried out in the future to further investigate these contrasting results in various surveys. Our results also illustrate that the marital status of participants was significantly associated with infertility knowledge, with single individuals having a higher mean knowledge score than married and divorced participants, which is in line with findings from another study [23].

To evaluate whether there is a relationship between income status and infertility, our study used vehicle ownership to assess the financial background of the participants and it was found that participants with more than one vehicle had a better mean knowledge score of infertility. Another study to evaluate the general health of infertile couples in Iran revealed that couples who belonged to a low-income background reported worse general health scores as compared to couples belonging to a high-income background [24]. These results highlight that poor infertility knowledge, low overall health, and not seeking timely treatment for infertility are prevalent in low-socioeconomic status. Thus, higher education and higher societal class predict higher knowledge levels regarding infertility, as sexual health education is not covered by most public school and college syllabi thereby depriving most youth of this integral knowledge during their formative years.

The findings of this study should, however, be interpreted in light of some limitations. The collection of data from a single site and the use of convenience sampling technique introduce the possibility of the study population not being a true representation of the community at large. Moreover, the method used here to gage the participants' financial background via the number of vehicles owned has not been validated by previous studies, and a more comprehensive measure (e.g. annual salary) should be used in future studies to accurately ascertain this information. Lastly, further studies must be carried out on a larger more diverse population in order to validate these findings. 

## Conclusions

Our study results reveal that awareness regarding infertility and its causes is lacking. Knowledge of infertility and its causes must be spread amongst individuals in Pakistan so that their misconceptions can be clarified and treatment if needed, be sought on time. Media such as television and newspapers should be utilized to impart knowledge. This would further help in identifying specifically the deficient areas of knowledge [such as the role of male factor, pelvic inflammatory diseases (PIDs)] that need to be emphasized on during outreaches and awareness campaigns. These findings would be a great asset in the development of leaflets and brochures to be given out at primary and specialized healthcare (OBGYN) clinics, for the education of the visiting patient population.
